# Usability and Acceptability of ASSESS MS: Assessment of Motor Dysfunction in Multiple Sclerosis Using Depth-Sensing Computer Vision

**DOI:** 10.2196/humanfactors.4129

**Published:** 2015-06-24

**Authors:** Cecily Morrison, Marcus D'Souza, Kit Huckvale, Jonas F Dorn, Jessica Burggraaff, Christian Philipp Kamm, Saskia Marie Steinheimer, Peter Kontschieder, Antonio Criminisi, Bernard Uitdehaag, Frank Dahlke, Ludwig Kappos, Abigail Sellen

**Affiliations:** ^1^ Microsoft Research Human Experience & Design Cambridge United Kingdom; ^2^ Neurology, Department of Medicine, Clinical Research, Biomedicine and Biomedical Engineering University Hospital Basel Basel Switzerland; ^3^ Global eHealth Unit Imperial College London London United Kingdom; ^4^ Novartis Pharma AG Basel Switzerland; ^5^ VU University Medical Center Amsterdam Netherlands; ^6^ Department of Neurology University Hospital Bern Bern Switzerland; ^7^ Microsoft Research Machine Learning and Perception Cambridge United Kingdom

**Keywords:** depth-sensing computer vision, information interfaces and presentation, Kinect, motor skills, multiple sclerosis, rehabilitation

## Abstract

**Background:**

Sensor-based recordings of human movements are becoming increasingly important for the assessment of motor symptoms in neurological disorders beyond rehabilitative purposes. ASSESS MS is a movement recording and analysis system being developed to automate the classification of motor dysfunction in patients with multiple sclerosis (MS) using depth-sensing computer vision. It aims to provide a more consistent and finer-grained measurement of motor dysfunction than currently possible.

**Objective:**

To test the usability and acceptability of ASSESS MS with health professionals and patients with MS.

**Methods:**

A prospective, mixed-methods study was carried out at 3 centers. After a 1-hour training session, a convenience sample of 12 health professionals (6 neurologists and 6 nurses) used ASSESS MS to capture recordings of standardized movements performed by 51 volunteer patients. Metrics for effectiveness, efficiency, and acceptability were defined and used to analyze data captured by ASSESS MS, video recordings of each examination, feedback questionnaires, and follow-up interviews.

**Results:**

All health professionals were able to complete recordings using ASSESS MS, achieving high levels of standardization on 3 of 4 metrics (movement performance, lateral positioning, and clear camera view but not distance positioning). Results were unaffected by patients’ level of physical or cognitive disability. ASSESS MS was perceived as easy to use by both patients and health professionals with high scores on the Likert-scale questions and positive interview commentary. ASSESS MS was highly acceptable to patients on all dimensions considered, including attitudes to future use, interaction (with health professionals), and overall perceptions of ASSESS MS. Health professionals also accepted ASSESS MS, but with greater ambivalence arising from the need to alter patient interaction styles. There was little variation in results across participating centers, and no differences between neurologists and nurses.

**Conclusions:**

In typical clinical settings, ASSESS MS is usable and acceptable to both patients and health professionals, generating data of a quality suitable for clinical analysis. An iterative design process appears to have been successful in accounting for factors that permit ASSESS MS to be used by a range of health professionals in new settings with minimal training. The study shows the potential of shifting ubiquitous sensing technologies from research into the clinic through a design approach that gives appropriate attention to the clinic environment.

## Introduction

### Overview

The recent development of robust depth-sensing cameras for practically tracking human motion is being rapidly exploited for the assessment and rehabilitation of motor dysfunction. Depth-sensing cameras, such as Microsoft Kinect (Microsoft, Redmond, WA, USA), capture video images in which each pixel has a three-dimensional position. Processed by advanced computer vision and machine-learning algorithms, depth videos enable the quantification of human movement without the need for marker-based motion capture or gait analysis systems, which are both expensive and cumbersome [1]. A particular advantage is that nothing needs to be attached to the patient.

Depth sensing has been used to build a range of health care applications, including touchless interaction during surgical image navigation[2], movement rehabilitation for children with cerebral palsy [3], and improving cognitive performance in elderly people [4]. Within the domain of multiple sclerosis (MS), depth sensing has been used to improve posture during exercise for patients with MS [5] and incorporated into an exercise game (telerehabilitation system) to encourage balance and sensory integration [6]. Virtual reality games have also been used to motivate motor rehabilitation exercises [7].

The use of depth sensing has only recently been extended to the assessment of motor dysfunction. Although sensing technology has the potential to increase the reliability and validity of assessment compared with human observers [8], achieving the high levels of system accuracy required for diagnostic purposes has proven challenging. Research has mainly focused on the validation of Kinect against other objective measurement systems [9] and its ability to provide accurate measures for particular conditions [10,11]. Systems that successfully provide clinical assessment of motor ability with Kinect remain very much “in progress” [12].

### Background

ASSESS MS is being developed to support the assessment of motor dysfunction in people with MS. As a chronic inflammatory disease of the central nervous system, MS causes a variety of symptoms, either in combination or alone. These include numbness, reduction in motor strength, and cerebellar dysfunctions as well as cognitive decline. The disease course is most frequently characterized by relapses in which the affected person experiences neurological symptoms followed by extended periods of remission in which symptoms may improve. Over time, the disease can enter into a progressive phase in which a steady deterioration occurs. Approximately 15% of MS patients experience ongoing deterioration from disease onset [13].

The unpredictability of the disease course makes the ability to track MS particularly useful. The condition is currently assessed with a standardized rating instrument based on clinical examination, the Expanded Disability Status Scale (EDSS) [14]. Patients are asked to perform a range of functional exercises, including stretching out 1 arm to the side and then touching the nose (finger-nose test) or walking on a pretend tightrope (tightrope walking). These exercises are summarized into functional system scores and, together with the ability to walk, are scored on an ordinal rating scale, from EDSS 0 to EDSS 10. Examinations are usually performed on a yearly basis. Although the EDSS is a widely used and accepted outcome measure, it suffers from low intrarater and inter-rater reliability making disease tracking difficult [15]. The expertise required also makes it infeasible for health professionals other than neurologists to perform the examination.

**Figure 1:**
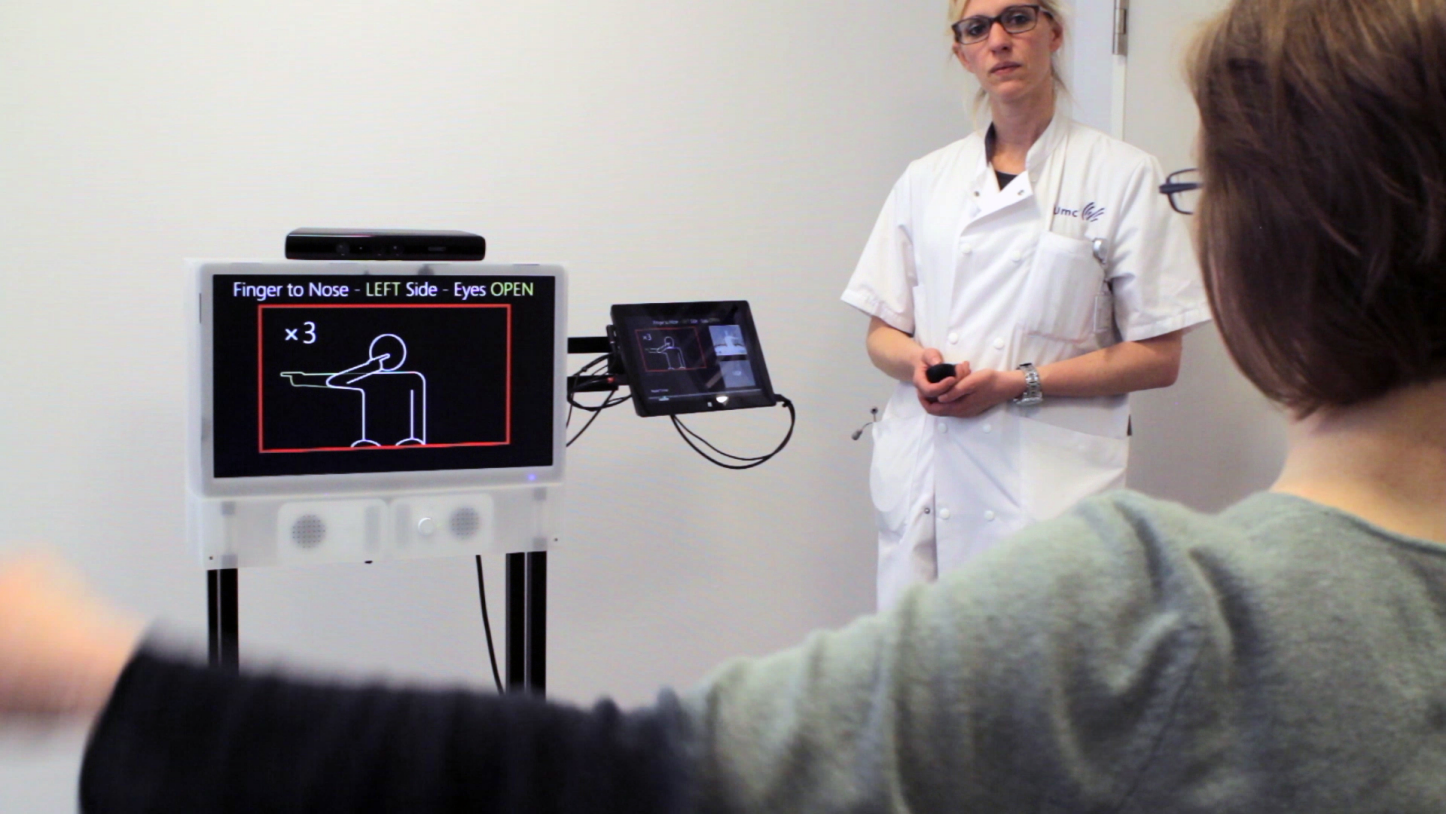
ASSESS MS being used to record a finger-nose test while the health professional monitors.

ASSESS MS aims to address this problem by quantifying changes in motor dysfunction more consistently and with finer granularity than currently possible. Shown in Figure 1, ASSESS MS captures depth videos of assessment movements with Kinect, which were performed by patients in a clinical setting with the support of the health professional. These are then processed to classify the severity of motor dysfunction. The level of accuracy needed for clinical assessment requires specific attention to the quality of the depth videos captured. Specifically, a high level of standardization is required.

The inherent unpredictability of hospital environments and the need for highly standardized data make the step from validating depth-sensing measures in the laboratory to creating a workable system in a clinical setting nontrivial [2]. Unintended variability, whether from inconsistent movement performance or poor image quality due to variable positioning or unexpected objects in the background, decreases the likelihood that the vision algorithms will be able to highlight variability that arises from disease. At present, there are no studies that show that this step is feasible for the clinical assessment of motor dysfunction with depth-sensing computer vision.

A key element of ASSESS MS is the design of both the physical device and the software application to support high-quality data capture in the clinical environment. The study presented here is a mixed-methods empirical evaluation of the usability and acceptability of these aspects of ASSESS MS. It aims to answer the following questions:

- Is ASSESS MS usable by health professionals?
- Is ASSESS MS acceptable to patients and health professionals?
- Are there any differences between neurologists and nurses in any of the metrics captured?

## Methods

### System Development

ASSESS MS was developed by a multidisciplinary team of researchers in human-computer interaction, machine learning, and health professionals caring for patients with MS. A 4-stage, iterative development process was undertaken to develop the physical device and software application used to support the recording and analysis of movements. Its design drew on multidisciplinary team meetings, design activities, observation of current clinical assessment practice, and user testing with healthy volunteers and patients. We first present the problems noted in the early stages of the project and the resulting design requirements before presenting the final system design. Algorithmic development is reported elsewhere [16].

### Design Requirements

Observation of clinical routines identified several issues that needed to be addressed. We observed that a neurologist could instruct the same movement in different ways. The finger-nose test, for example, might begin by stretching the hand out to the side and in other cases stretching the hand to the front before touching the nose. Clinicians were also observed to adapt the instructions given to a patient according to their abilities. For example, while able patients might be asked to bicycle their legs to assess strength, patients with a degree of disability would be asked to push their leg against the clinician’s hand.

This kind of variation, although of no consequence to a neurologist, is problematic for machine-learning algorithms, which statistically evaluate patients against “known” characteristics derived from training examples. If a large amount of variation in movement performance arises from factors other than those relating to disease state, classification ability inevitably decreases. The findings emphasized the need to not only provide cues to standardize the movement, but also offer discretion for health professionals to omit movements if necessary, as well as repeat them if performed incorrectly.

We also noted that current examinations are an embodied interaction between clinician and patient. It was not uncommon for a clinician to stand in front of a patient and demonstrate the movement to be performed. A clinician might also touch the patient to indicate how to do a movement or which side to use. Most importantly, the clinician may have to stand next to a patient due to safety reasons. Many of these typical interactions had the potential to disturb the image captured by ASSESS MS, either by blocking the camera view or creating a challenge to distinguish between patient and doctor. At the same time, appropriate patient-health professional interactions are important to ensure patients feel safe and cared for.

Not least, the clinical examination is a mobile affair. Patients can move large distances while performing a movement, such as hopping on 1 foot. Patients may perform small movements, adjusting their sitting position, which can lead to limbs being out of the camera view. In smaller rooms, furniture needs to be adjusted and the camera moved multiple times between different types of tests (eg, sitting and standing tests), which can change the camera view and the placement of the patient in that view. With all of this movement, it was necessary to achieve as standard a lateral and depth positioning as possible, to facilitate the preprocessing of the videos.

This initial work suggested that ASSESS MS needed to support the following aspects of capture:

- Standardized movement instruction;
- Flexible interface for the health professional to record movements;
- Facilitated patient-health professional interaction with maintained image quality; and
- Precise positioning of the patient.

### ASSESS MS Description

**Figure 2:**
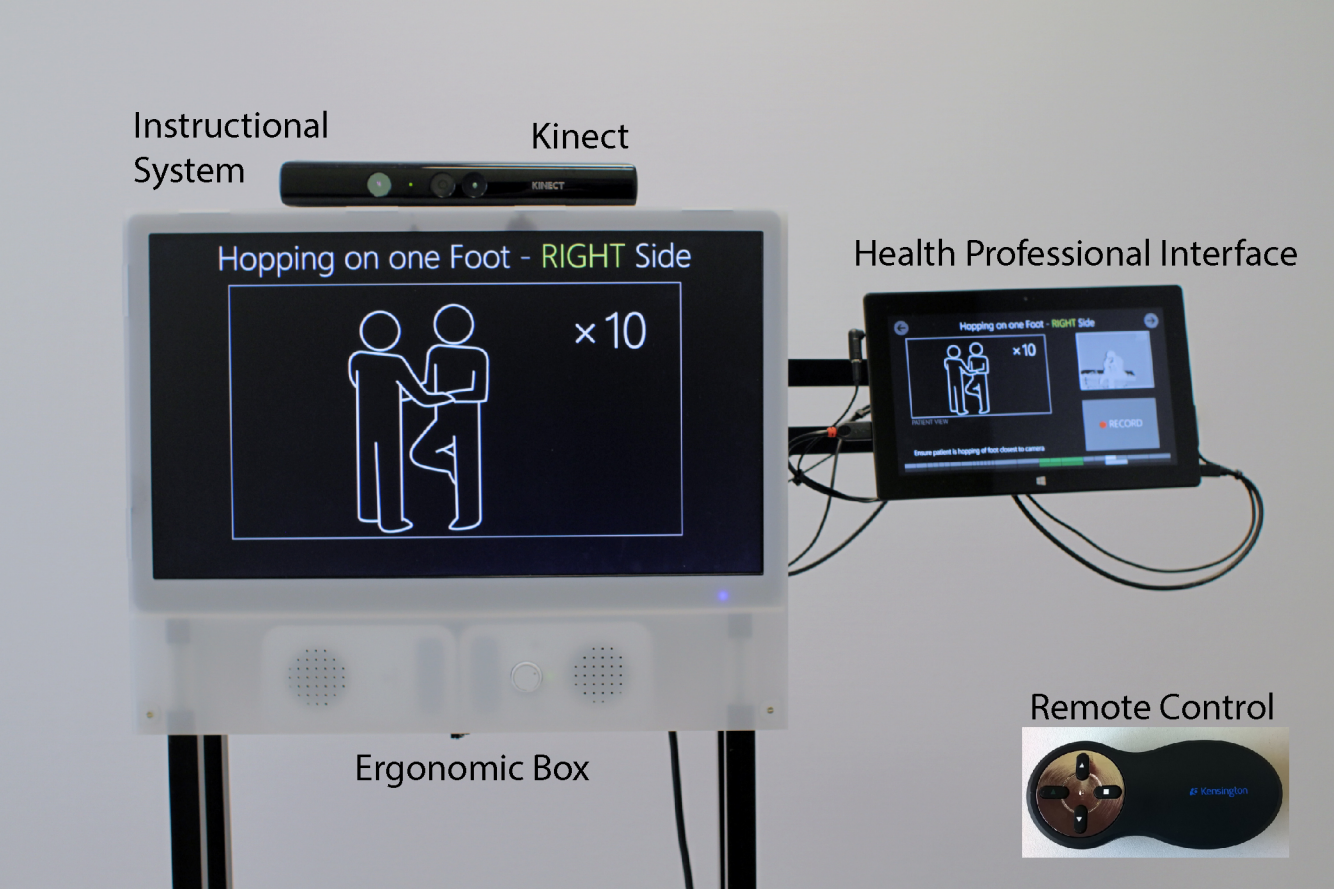
Elements of ASSESS MS, including instructional system, Kinect depth-sensing camera, health professional interface, remote control, and ergonomic box.

ASSESS MS, shown in Figure 2, has a 53.3-cm (21-in) patient-facing screen used to instruct patients in the assessment movements. A smaller tablet computer with touch-screen capability is mounted on a mobile arm at the back of the unit. This interface is used by the health professional to position the patient, select the assessment movements to be performed, and complete the recordings. A remote control enables the health professional to move freely around the room to support the patient as needed. The screens sit in an ergonomic box on wheeled legs for ease of maneuvering with the Kinect mounted on top.

**Figure 3:**
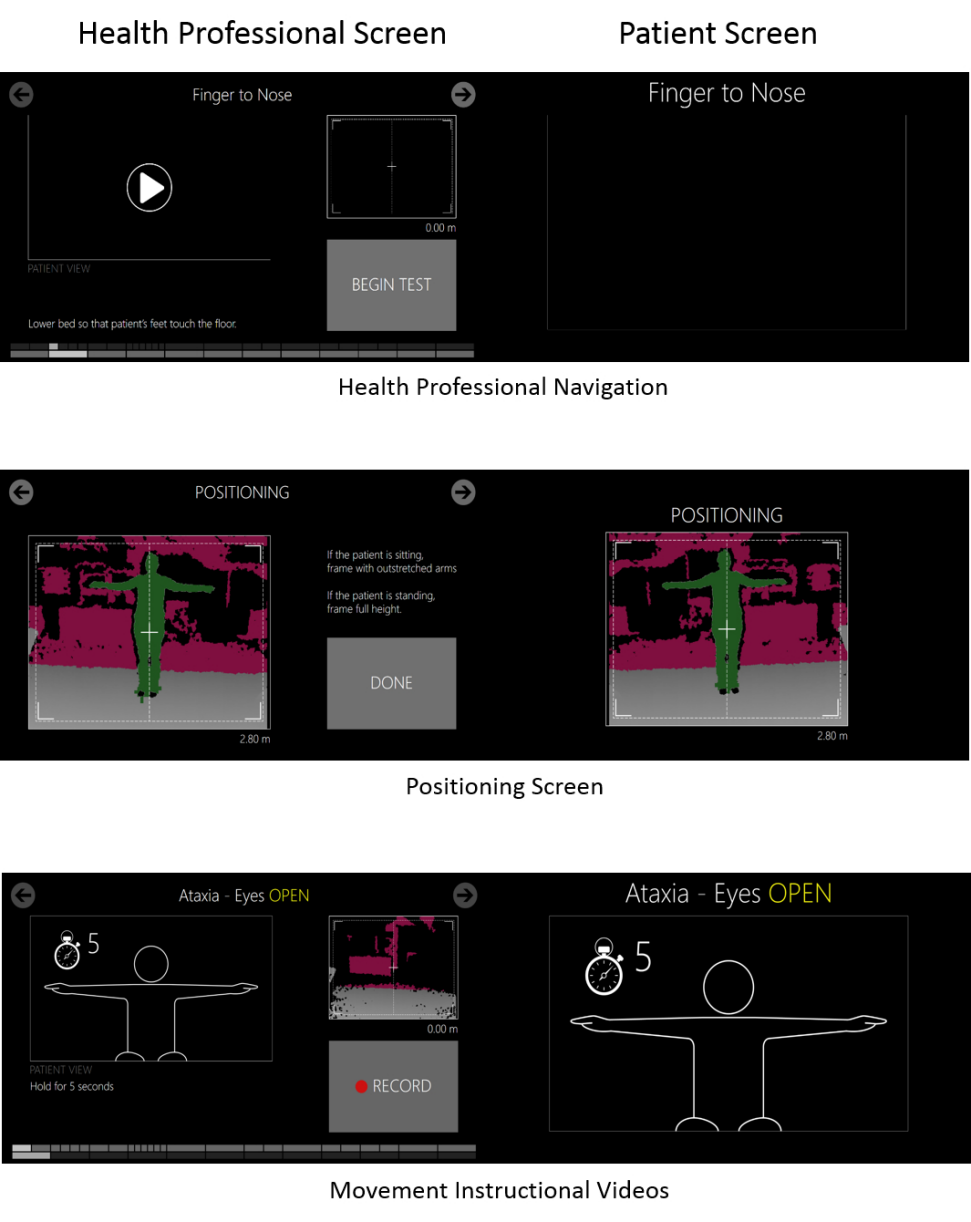
Screenshots of interface: health professional screen on the left and patient screen on the right. Top, navigational elements; middle, positioning feature; bottom, movement instructional videos.

The health professional interface provides a number of navigational options. The health professional can play a movement instruction video, or begin a test. Arrows at the top of the interface enable the health professional to skip movements (eg, finger-nose test) or variations of movements (eg, left side, eyes open). Movements can be repeated by skipping backward. Each page contains a button, which enables the beginning of a test, recording of a movement, or stopping of a recording. A navigational bar at the bottom shows visually which movements have been captured and which skipped. These are all shown in the top image of Figure 3.

Precise support for positioning is provided through an easily maneuverable device used in conjunction with the “positioning” feature, as shown in the middle image of Figure 3. This screen provides a view of the depth image stream with a center crossbar to which the patient should be aligned. It is available before the sitting and standing components in the test as a full-screen feature and in a persistent window in the upper-right-hand corner throughout. The distance of the person from the camera is indicated below the image. It was intended to reduce variability in positioning.

Movement instructional videos are provided to standardize movement performance. They guide the patient, as well as the health professional, about exactly how to perform the requested movement. They consist of simple line drawing animations accompanied by verbal descriptions localized into 3 languages. The design of the animations was based on the psychology literature on movement learning, which emphasized simplicity of representation [17] and the importance of drawing attention to the most distal point of movement, for example, the hand when moving the arm [18].

A number of approaches were taken to support the patient-health professional interaction in light of the instructional videos that change the nature of this interaction. First, the placement of the health professional interface is intended to encourage health professionals to stand to the side or back of the device to avoid blocking the camera view. Second, automatic recording of movements was not used to enable appropriate pauses in the examination to facilitate interaction.

### Movement Protocol

**Figure 4:**
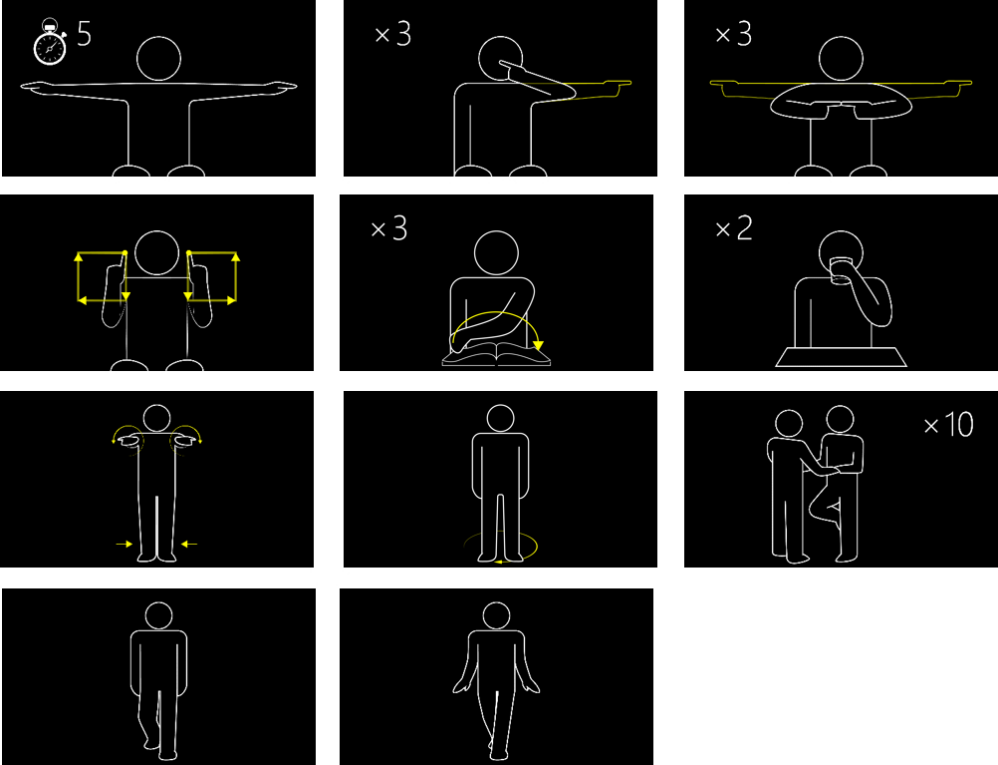
The 11 assessment movements in the ASSESS MS movement protocol: truncal ataxia, finger-nose test, finger-finger test, drawing squares, turning pages, drinking from cup, Romberg test, turning on the spot, hopping on 1 foot, normal walking, and tightrope walking.

The movement protocol contained 11 movements depicted in Figure 4. Of these 11 movements, 6 were chosen from the EDSS examination to cover the function of the upper and lower extremities and the trunk. Two activities of daily living movements, drinking from a cup and turning pages in a book, were also included. In addition, 3 new movements were defined, which included finger-finger test, drawing squares, and rotating on the spot. These were created to capture the upper and lower extremity functions in a potentially more camera-friendly way.

### Study Design

Health professionals, previously unfamiliar with ASSESS MS, were asked to use it to examine 4 MS patients following 1 hour of training. The training covered the importance of standardized movement performance, the movement protocol, and the features to promote image quality. A “cheat” sheet was given to all of the health professionals with the details of the movement protocol for ease of reference (see Multimedia Appendix 1). The set of examinations done by a given health professional took place within a week, and most on a single day.

Patients were included if theyhad a diagnosis of MS and an EDSS score between 0 and 7. After giving informed, written consent, patients were randomly assigned to a health professional. To minimize inconvenience to patients, invitations to act as participants were extended to individuals already attending routine clinic appointments during the study period. Some patients were given an examination with the same movement protocol [16], within a parallel ongoing trial (n=10), but none within the previous 3 months. Ethical approval was obtained in all 3 hospitals.

### Outcomes

*Usability* has been defined by the International Organization for Standardization in ISO 9241-11 [19] as “the extent to which a product can be used by specified users to achieve specified goals with effectiveness, efficiency and satisfaction in a specified context of use.” In this case, we wanted to achieve the following 4 goals:

1. Complete a full protocol of recordings, repeating, or skipping movements as necessary.
2. Obtain standardized movement performance from patients.
3. Position ASSESS MS adequately for quality data capture.
4. Ensure the camera view is not blocked by the health professional.

The *context of use* is defined as a clinical setting.

We focused on 2 types of *users* (neurologists and nurses). While neurologists are the group that currently performs neurological examinations in clinical trials, it would be more cost effective if semiautomated examinations could be completed by other health professionals. These might include nurses, study nurses, or paramedic staff. As most will be nurses, we refer to them hereafter as such. Recognizing this range of potential users, ASSESS MS was deliberately designed for nonexperts with minimal training.

The way that the ISO definition of usability is tested depends very much on the technology. We have articulated specific metrics for effectiveness, efficiency, and satisfaction as detailed in Table 1 that match the goals of the system and context of use specified earlier.

*Acceptability* is part of the ISO definition of usability under the term “satisfaction.” However, we take a view of acceptability more consistent with studies in the clinical domain. We assume that system acceptance is the trade-off between the benefit provided and any discomfort that arises, rather than some inherent good feeling that is gained. It is measured through willingness for future use, impact on patient-health professional interaction, and general perceptions of the technology as shown in Table 1.

**Table 1:** Description of metrics used for each trait and the data source drawn upon.

Trait^a,b^	Metric	Data source
Effectiveness	Task completion	Video
	Movement performance standardization	Depth recordings
	Adequate positioning	Depth recordings
	Clear camera view	Depth recordings
	Clarity of task	Questionnaire (Q1 and Q3)
Efficiency	Time to completion	Video
	Perceived efficiency	Interviews
Satisfaction	Covered by acceptability	
Future use	Willingness to use again	Questionnaire (Q2)
Interaction	Attitudes toward human interactions	Questionnaire (Q4 and Q5)
	Articulated changes to patient-health professional interactions	Interviews and video
Perceptions	Description of recording tool and associated issues	Patient questionnaire (Q6-Q9) and interviews

^a^Effectiveness, efficiency, and satisfaction constitute “usability.”

^b^Future use, interaction, and perceptions constitute “satisfaction.”

### Data Collection

In addition to the depth recordings taken as part of the ASSESS MS examination, all assessments were video recorded with a separate digital video camera that captured the way health professionals and patients interacted in the examination room.

A 9-item questionnaire was given to patients at the end of the examination. It contained 5 Likert-scale-type questions and 4 free response questions. The Likert-scale questions were developed by researchers in human-computer interaction (CM and AS) based on 2 key usability constructs: ease of use and impact on human interaction. Q1 and Q3, respectively, queried ease of use of the whole system and its instructional aspects alone. Q5 and Q4 queried the same for impact on human interaction. Q2 focused on the acceptability of ASSESS MS as determined by willingness for future use. Positively and negatively framed questions were counterbalanced.

Four open-end questions were asked to enable an opportunity for patients to give a more extensive commentary on their experience. The first asked patients to characterize ASSESS MS for another patient in 3 words. The second and third focused on the most and least helpful aspects of ASSESS MS. The fourth and final question provided space for any further comments. Questionnaires were translated into German and Dutch by the respective clinical teams, and wording for this translation was agreed internally. Local piloting with patients was carried out in 1 clinic in which multiple clinician input could not be obtained.

Health professionals were given a similar 5-item Likert-scale-type questionnaire after each examination. The questions were intended to be equivalent to the patient questionnaire for comparative purposes. Following the completion of questionnaire by all patients, professionals also took part in a 15-minute debriefing interview. The questions were similar to the free response questions given to the patients, but done in an interview form to enable more extensive discussion (see Multimedia Appendix 2).

To assess whether the results applied to a wide spectrum of patients, the EDSS and the Symbol Digit Modalities Test (SDMT) scores were recorded for each patient. Calculated from a detailed neurological examination, the EDSS was used to assess physical disability. The scale includes 20 half steps, ranging from 0 (normal) to 10 (death due to MS) [14]. The SDMT was used as a measure of cognitive ability. It examines information processing speed, visual working memory, and concentration by primarily assessing complex and visual scanning and tracking. It ranges from 0 (no correct answers) to 110 (all correct answers) [20].

A technology researcher (KH) was present throughout the study to manage the questionnaires and interviews as well as any technical issues that arose. This person did not intervene in the conduct of examination itself. A clinical researcher (MDS, JB, SMS, or CPK) was also available for patient or health professional queries.

### Data Analysis

Videos were coded for task completion. A task was completed if all recordings were made, repeating and skipping movements as necessary, without intervention from the researcher. Support for system crashes was not counted as intervention because ASSESS MS is still at the prototype stage.

The length of examination was calculated for the first and final patients of each health professional. “Start time” was defined by the first keystroke of entering patient information and “end time” by the completion sound generated at the end of the final test, rounded to the nearest 5 seconds. Crashes were subtracted from the overall time from the moment the health professional realized there was a crash to the time at which he/she was able to resume. If the first or final patient was wheelchair bound or had severe cognitive decline identified by the health professional which changed the length of the examination substantially (because multiple movements were not performed), the next examination was used instead.

To test the statistical difference between length of examination of first and final patients of a health professional and between neurologists and nurses, we used Student *t* tests. Three videos could not be coded due to recording errors, such as missing beginning or video camera pointing in the wrong direction.

A metric of standardized movement performance was calculated from review of the depth videos. All sitting movements were scored for correct directionality of movement, for example, finger-nose test was performed with the arm to the side rather than front, and the correct number of repetitions (see Multimedia Appendix 3). Standing movements were not rated due to poor-quality images and/or preprocessing elements (eg, head detection) not yet available. Two people (CM and her colleague) rated 27 examinations, sampled to exclude the first 2 examinations by the participating health professionals, which were treated as training cases. Disagreements were resolved through discussion. Whether the camera view was clear was coded at the same time as the patient being visible throughout the entire examination.

The metric for adequate positioning was derived mathematically during the preprocessing of the depth videos before their usage in the machine-learning algorithms. In the case of lateral positioning, we report the number of pixels needed for the head to be transposed for it to be centered in the image. We considered anything within half the diameter of the “average” head size as “good positioning,” as this would enable easy head detection. The “average” head size in our sample was 56 pixels. It was calculated by measuring the segmented area on 100 randomly picked processed finger-nose test videos at one fourth of the image height from the top. This should equate with about the eye level. For depth positioning, we report distance from that specified in the movement protocol. Any value within the diameter of the “average” head size (18 cm) was labeled as good positioning. This approach accounted for natural movement of the head.

The Likert-scale questionnaire data were tabulated and descriptive statistics were used. We considered answers per site as well as answers in aggregate; Student *t* tests were used to compare between sites. One patient’s questionnaire data were removed, as this patient provided the same answer for all questions, suggesting a lack of attention to the questions. As the questions were balanced, both positive and negative answers would be expected. The responses to the free response questions (Q7-Q9) were minimal with only 46.4% (71/153) containing answers. The majority of answers contained only a few words that were prosaic in nature, for example,“Interesting system.” These have been included in the reporting of the interviews when applicable but, for the most part, contributed little to the data analysis.

The words from the first free response question asking for 3 words to describe ASSESS MS to another patient were grouped into the following 3 categories: positive (eg, interesting), negative (eg, slow), and characteristic (eg, computer system). To gain a more nuance view of how the patients viewed ASSESS MS, a researcher (CM) and 2 visual designers grouped the words provided into a word cloud. These were melded into a visualization by an experienced visual designer. Words that are larger were those repeated more often (the number is shown in the visualization). Words on opposite sides of a line were considered contrasting. No words were deleted from the visualization so that the viewers might interpret for themselves the kind of language used to describe ASSESS MS.

The health professional interviews were coded for implicit or explicit discussions of standardization, patient-health professional interaction, and system comments. These themes were chosen after a first listening of the interviews as they encompassed the data, while providing responses to important design decisions. The interviews were further understood by viewing the associated examination videos after listening to the interviews.

## Results

### Participants

We recruited a convenience sample of 12 health professionals. Half were neurologists and half nurses. Participating health professionals were evenly split across 3 hospital sites in 2 countries. Their specialty and years of experience are presented in Table 2.

A total of 51 patients were recruited to the study. Slight over-recruitment (proposed sample n=48) was intended to address dropout due to patients choosing not to participate on the day; however, all patients recruited participated. Patients spanned a wide range of levels of physical and cognitive disability, as well as age, as seen in Table 3.

**Table 2:** Health professional characteristics.

Characteristics	Neurologists (n=6)	Nurses (n=6)
Age, mean (range), years	36.5 (26-53)	43.8 (27-61)
Gender (female/male)	4/2	6/0
Experience with MS, mean (range), years	4.3 (0.5-15)	2.7 (0.5-5)
Experience with physical examination, mean (range), years	3.4 (0.5-15)	0.8 (0-4)
Professional status	1 consultant, 2 attending physicians, 2 residents, and 1 medical student	4 study nurses, 1 clinical epidemiologist, and 1 study coordinator

**Table 3:** Study patient characteristics.

Characteristics	Total patients(n=51)
Age, mean (range), years	46.0 (23-73)
Gender (female/male)	31/20
Disease duration, mean (range), years	14.2 (0.5-47)
Disease course (CIS/RRMS/SPMS/PPMS)^a^	1/37/7/6
EDSS, median (range)	3 (1-7)
Symbol Digit Modalities Test, median (range)	47 (13-79)

^a^CIS = clinically isolated syndrome; PPMS=primary progressive MS; RRMS=relapsing remitting MS; SPMS=secondary progressive MS.

### Usability

#### Effectiveness

All health professionals were able to carry out the examination appropriately using ASSESS MS without guidance after the first examination. This included positioning ASSESS MS, playing the instructional videos, capturing recordings, or navigating to different tests and subtests to repeat or skip a movement. No consistent task errors were identified by either researcher (CM/KH) involved in the analysis. Movements were performed by patients according to the protocol in 97.6% (405/415) of the cases. Most mistakes occurred in the drawing squares movement, but were not attributable to specific patients or health professionals. The camera view was never blocked. As illustrated in Table 4, no differences were seen across clinics in any metric. There was no substantive difference between neurologists and nurses.

**Table 4:** Metrics of usability (effectiveness) for each clinic and the aggregate.

Metric(patients)	Clinic 1(n=17)	Clinic 2(n=18)	Clinic 3(n=16)	Total(n=51)	Percentage
Task completion	17/17	18/18	16/16	51/51	100
Standardized movement performance	142/143	150/155	113/117	405/415	97.6
Clear camera view	17/17	18/18	16/16	51/51	100

**Figure 5:**
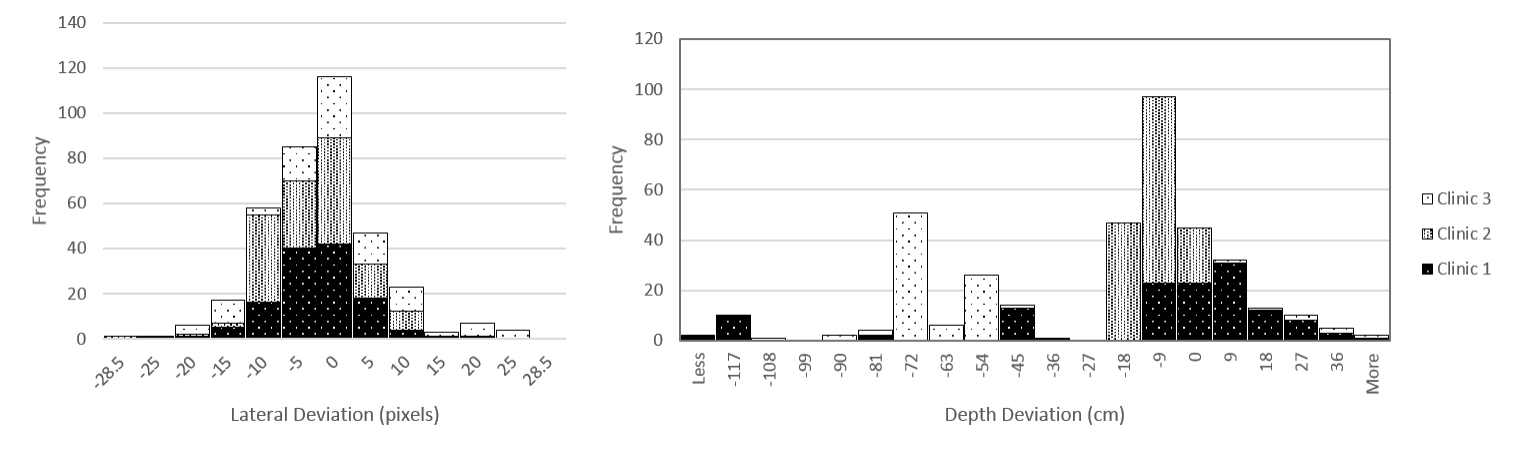
Histogram of frequency of deviation from normalized point. Lateral deviation is presented in pixels (left) and depth deviation in centimeter (right).

Patients were laterally positioned consistently, with only 1 (n=368) outside the 28.5-pixel margin. There was less consistency in the distance between recording tool and patient, with 134 (n=368) being more than the 18 cm from the normalized distance. There was a substantial skew of videos being farther away than the requisite distance, as shown in Figure 5.

The questionnaire data indicate that both patients and health professionals gave high scores for ease of use as well as clarity of instruction as shown in Table 5. Patients gave higher ratings than health professionals. That said, many of the health professionals interviewed highlighted that the clear instructions given by the recording tool and minimal instruction given by the health professional should be effective in capturing data. As one health professional said:

“The system makes it easier to explain to the patient and they are more likely to do it correctly.” (HP7: Doctor)

There were some differences between clinics, with Clinic 1 having significantly lower (*P*<.001) ratings than Clinic 2 by health professionals.

**Table 5:** Questionnaire data for effectiveness metric.

		Clinic 1	Clinic 2	Clinic 3	Total	Ideal^a^
Patients						
	I understood what to do during the study examination, mean (SD)	6.3 (1.3)	6.8 (0.4)	6.7 (0.5)	6.6 (0.9)	7
	The movement instructions given by the recording system were clear, mean (SD)	6.1 (1.4)	6.6 (0.6)	6.6 (0.5)	6.4 (1.0)	7
Health professionals						
	The recording system was easy to use, mean (SD)	5.0 (1.1)	6.7 (0.6)	5.4 (1.6)	5.7 (1.4)	7
	The movement instructions given by the recording system were clear to the patient, mean (SD)	5.1 (1.4)	6.2 (0.9)	5.7 (1.2)	5.7 (1.2)	7

^a^1 indicates “strongly disagree” and 7 indicates “strongly agree” (Likert scale).

#### Efficiency

The mean time to completion for a health professional’s final examination was 18:59 (mm:ss). There was a significant decrease in average recording time between the first and final examination times (*P*<.001), as shown in Table 6. This suggests that efficiency increases quickly with minimal experience. There were no significant differences (First examination: *P*=.55; Last examination: *P*=.91) in the length of examination between neurologists and nurses.

The recording tool was specifically highlighted as efficient by a number of health professionals. There was no comment on it being inefficient. As one health professional said,

“It is quick because it is so structured. What often happens is that the patient starts explaining things and talking about problems and it takes ways longer. If you follow it, you’re finished in 15-20 minutes. It keeps the focus on what matters.” (HP12: Nurse)

**Table 6:** Mean examination length of the first and final examinations of all health professionals and of nurses and doctors separately.

	First examinationMean (SD) time (mm:ss)	Final examinationMean (SD) (mm:ss)
Health professionals	26:55 (04:33)	18:59 (02:50)
Doctors	27:45 (04:28)	18:52 (03:50)
Nurses	26:06 (04:53)	19:05 (01:41)

### Acceptability

#### Future Use

Patients disagreed with the statement that they would not like their health professional to use this system in future suggesting that it would be acceptable to use. Health professionals had a more varied view, but with aggregate scores that would still suggest acceptability (Table 7). One health professional opposed future use and gave very low scores. With no examination experience, health professionals found it challenging to manage both the patient and the technology, despite completing the recordings successfully.

**Table 7:** Questionnaire data for acceptability metric (future use).

		Clinic 1	Clinic 2	Clinic 3	Total	Ideal^a^
Patients						
	I would not like my health professional to use the recording system during my future examinations, mean (SD)	2.5 (1.7) (n=17)	2.0 (2.0) (n=18)	1.5 (1.5) (n=16)	2.0 (1.8) (n=51)	1
Health professionals						
	I would use the recording system in future examinations, mean (SD)	4.1 (1.4) (n=4)	6.8 (0.7) (n=4)	4.8 (1.2) (n=4)	5.2 (1.6) (n=12)	7

^a^1 indicates “strongly disagree” and 7 indicates “strongly agree” (Likert scale).

#### Human Interaction

The recording tool did not make either patients or health professionals feel awkward. The highest ambivalence came from patients and health professionals with regard to the instructional aspects of the recording tool. These 2 questions had the highest level of variability with a greater number of patients providing a neutral answer (Table 8).

**Table 8:** Questionnaire data for acceptability metric (interaction).

		Clinic 1	Clinic 2	Clinic 3	Total	Ideal^a^	
Patients							
	I prefer my health professional to demonstrate the movements, mean (SD)	2.5 (1.8) (n=17)	1.6 (1.2) (n=18)	2.7 (1.7) (n=16)	2.3 (1.6) (n=51)	1	
	The recording system made me feel awkward or uncomfortable, mean (SD)	1.8 (1.3) (n=17)	1.8 (1.7) (n=18)	1.1 (0.3) (n=16)	1.6 (1.4) (n=51)	1	
Health professionals							
	I prefer to demonstrate the movements to the patient myself, mean (SD)	3.7 (1.7) (n=4)	2.3 (1.6) (n=4)	4.1 (1.0) (n=4)	3.4 (1.7) (n=12)	1	
	The recording system made me feel awkward or uncomfortable, mean (SD)	2.1 (0.2) (n=4)	1.4 (0.8) (n=4)	2.4 (1.4) (n=4)	2.0 (1.0) (n=12)	1	

^a^1 indicates “strongly disagree” and 7 indicates “strongly agree” (Likert scale).

Early testing of the prototype with neurologists suggested that some felt that ASSESS MS usurped their role, as articulated in the following quotation, and might pose an issue to acceptability:

“Usually everything that the computer tells the patient is something that you tell the patient as a physician, so the interaction is somehow reduced because you let the computer talk...I am used to tell the patient exactly what I want them to do so I can see what I want to see. There is nothing a physician needs to do. It is something that my assistant could do.” (Doctor)

We found similar sentiments in this study as well, formulated in different ways. One neurologist reflected on the feeling of loss of the physical connection with the patient that would normally be gained through touching the patient during the examination. Another neurologist discussed the disruption to her rhythm, saying “I have my rhythm, an interaction with just me and the patient. Here we have the third component...It’s a threesome.” A third neurologist spoke about an inability to move freely about the room as ASSESS MS occupied the limited floor space. A fourth neurologist spoke about the loss of the creative process of medicine, suggesting that this test could be done by assistants.

Despite these initial discomforts mentioned, neurologists adapted quickly to engaging with the patient while using ASSESS MS. Several neurologists noted that they said more than they needed to in the beginning, but decreased their verbal speech with time. As one said,

“The last patients understood the instructions better so I did not say anything and they did it well.” (HP11: Doctor)

Others felt that an examination was more personal if they spoke more often.

“I think I explain a bit more than necessary...Sometimes I just repeat what has already been said [by ASSESS MS]. I think sometimes the patient would have been able to just understand it just by listening. I personally think that it is somehow more personal if I say it again or point out what could have been important.” (HP6: Doctor)

In all cases, the doctors spoke less over time, with many using substantial body language, such as exaggerated nods or smiles to replace verbal interaction. Reflection on the videos suggests that all doctors had found an examination rhythm by the third patient. This was often gained through pre-emptively instructing the patients in the aspects of the movements that needed to be standardized.

Nurses did not have the same feelings and raised no comparable comments about using ASSESS MS. They took a different perspective on the instructional videos. Most mentioned the dual role of the video in advising them as well as the patient as to what to do. This is illustrated by requests from 3 nurse participants to have protocol information verbally included in the movement descriptions, such as “With the feet on the floor, raise your arm out to the side...” The nurses easily found a balance between interaction with the patient and ASSESS MS.

#### Perceptions

**Figure 6:**
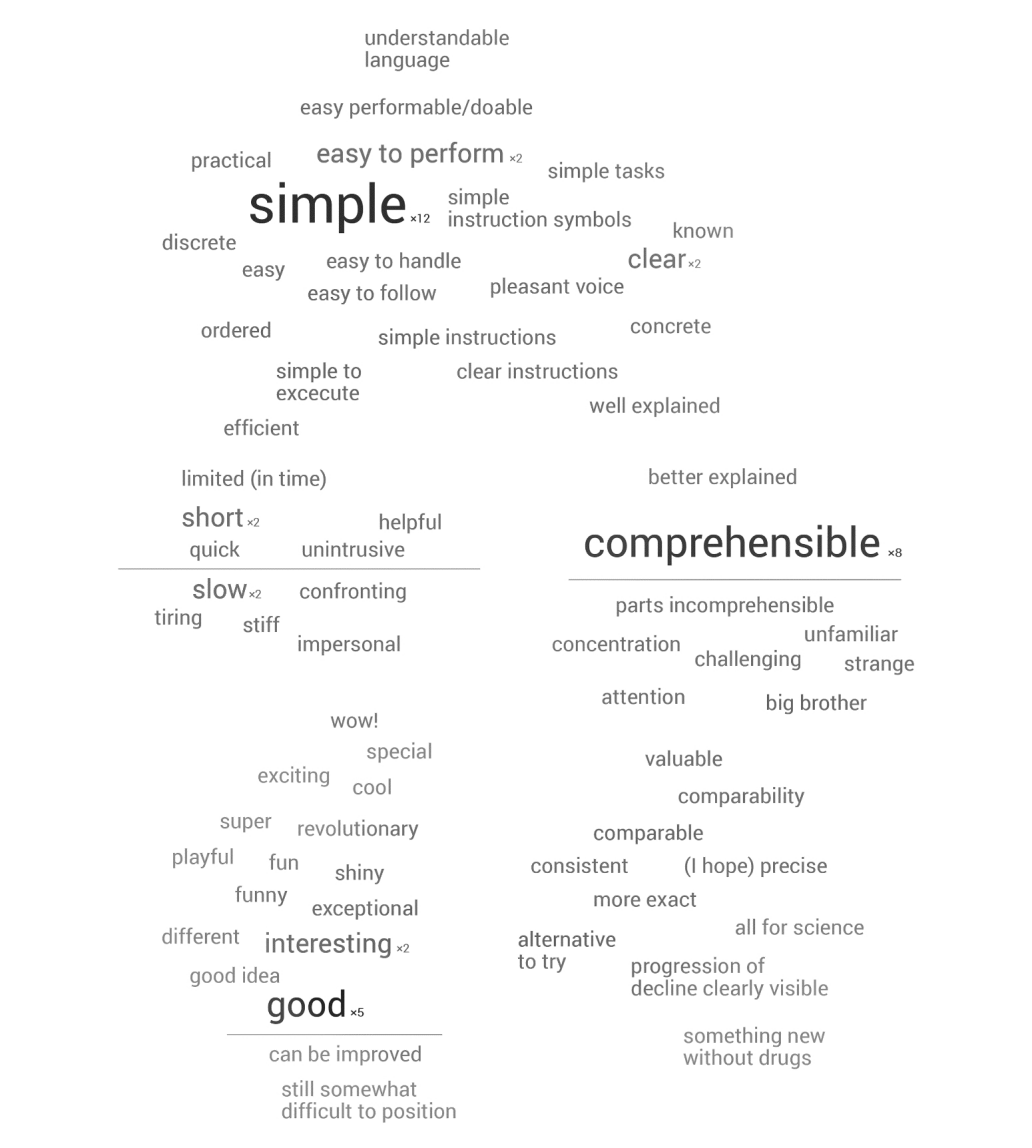
Word cloud of patients’ descriptive words of ASSESS MS.

Patient and health professionals were overwhelmingly positive when asked to give 3 words describing ASSESS MS. Upon tabulating the individual words patients used to describe ASSESS MS, 78 were found to be positive (eg, simple), 12 negative (eg, slow), and 29 characteristic (eg, computer system). For the health professionals, there were 19 positive, 2 negative, and 5 characteristic words. Figure 6 provides a visualization of all of the words used by patients to describe ASSESS MS. The feedback of the health professionals focused mainly on specific technical fixes (eg, the improved phrasing of a particular movement instruction). No negative feedback was given during the interviews.

## Discussion

### Principal Findings

The ASSESS MS is a system to support the assessment of motor dysfunction in patients with MS using depth-sensing computer vision. It aims to provide a consistent, quantified metric of motor ability to enable finer-grained tracking of disease progression than currently possible. ASSESS MS has been designed to facilitate more standardized data capture to support movement analysis by the machine-learning algorithms, while being both usable and acceptable by patients and health professionals in clinic settings. Our results show high levels of usability and acceptability by both patients and health professionals. There was little variation in results across the 3 participating centers, and no differences between neurologists and nurses.

The study suggests that ASSESS MS is usable. It is effective in that all health professionals were able to complete the recordings, with high levels of standardization of movement performance, lateral positioning, and clear camera view. It was also perceived as easy to use by both patients and health professionals with high scores on the Likert-scale questions and positive comments being provided during the interviews. The variation achieved in the patient population indicates that patients’ level of physical (EDSS range from 1 to 7) or cognitive (SDMT from 13 to 79) disability does not change the effectiveness of the tool.

The only aspect of ASSESS MS that was problematic was the distance positioning. Closer inspection of the videos suggested that distance was frequently gauged through physical landmarks in the room (eg, wall), rather than the distance provided on screen. Clinic 2, which had room furniture at the correct distances to facilitate alignment had the highest consistency of depth positioning, placing ASSESS MS in front of a bookcase for the sitting exercises and then against a desk for the standing exercises. This suggests that the physical properties of the room should be reviewed and highlighted in the training when a new site is trained to use ASSESS MS.

ASSESS MS also seemed to be reasonably efficient with the average time under 20 minutes and decreasing with use. Because the examination did not incorporate the complete EDSS, no direct comparison was possible. The lower bound of completion time, about 13 minutes, suggests that the test may be completed in less time with further emphasis on speed. That health professionals felt it was efficient is also important.

ASSESS MS was acceptable to patients on all dimensions considered, including future use, interaction (with health professionals), and perceptions. There was little variation of scores, with most clustered at one end of the Likert-scale and with a very few at the opposite end. This suggests that most people had high acceptability and a small number would not use it, but there was little ambivalence. There was no clear pattern of those who opposed it, for example, by age or level of disability. Variations in attitudes to new technologies, including negative perceptions, are predicted by models of staged technology adoption [21]. The word descriptions of ASSESS MS were overwhelmingly positive. These results strongly suggest that ASSESS MS is acceptable to patients.

Health professionals also accepted ASSESS MS, but with greater ambivalence. Clinic 1 seemed to have particularly low scores, which we attribute to experiencing the most technical issues, such as the disconnection of the Kinect camera feed from the application that required a restart by the supervising technology researcher (KH). It was interesting that the 2 professionals who were new to MS had no difficulty performing the test with movements that they did not know. Two other health professionals with little or no examination experience in any area of medicine, however, were more uncomfortable. Training in examination skills should also be provided for those without previous experience.

There were no differences between neurologists and nurses in any quantitative metric analyzed, including length of examination. The only difference lay in attitudes. Neurologists found it initially uncomfortable to work with this semiautomated, highly standardized system, whereas nurses welcomed the support the system provided. That said, neurologists used their examination skills to pre-empt potentially incorrectly performed movements building an interaction around achieving standardized movement performance. We would suggest that ASSESS MS is particularly well suited to health professionals other than neurologists, but is flexible enough to be used by any health professional.

These findings indicate that ASSESS MS in its current version is both usable and acceptable. It can be deployed to new sites and used by a range of health professionals with just 1 hour of training. This stands in contrast to current tools, such as the EDSS, which require a standardized training and an experienced background in clinical neurology. As such, ASSESS MS has the potential for inexpensive, widespread use.

### Anecdotal Lessons

The training process gave substantial insight into how health professionals came to understand ASSESS MS. It worked best to provide a simple characterization of how the machine learning worked as a process of comparison between new data and past patients that it had “seen.” This meant that if a patient “looked like” they had a given disability level, then they would be labeled as such, irrespective of whether that was because they actually had that level of dysfunction or because they did not perform a movement correctly. Providing this fairly simple account of how ASSESS MS works was motivating for the health professionals in trying to achieve standardized data.

We also found that a small change to the way the movement protocol was introduced, by asking health professionals to perform it as opposed to watch it, increased confidence. That said, the movement protocol was the most challenging part of the health professionals’ learning curve, and perhaps not captured in the system-oriented measures in this paper. There were numerous questions about how to perform the protocol and the availability of one of the clinical researchers to correct errors between examinations was welcomed. When considering training in future, the availability of such a person should be included, and the greater emphasis on the movements as opposed to system use should be considered.

### Limitations

Usability is only generally specified, with specific metrics required for each technology being assessed. We could have assessed many aspects of ASSESS MS, but have focused on ones that we think are critical to producing a workable system for both health professionals and data engineers. As a novel technology that aims to achieve something very different than the status quo, a direct comparative is not available. As such, what constitutes a “good” result can be disputed. It is difficult, for example, to provide an exact bound on what is acceptable positioning or a necessary percentage of standardized movement. That said, we know that these elements are essential to the system and want to continue increasing these numbers.

The choices made for measures matched the design criteria of ASSESS MS, but technology moves quickly. The original Kinect is no longer on the market, for example, and the machine-learning algorithms will continue to develop. Further, ASSESS MS focuses only on a subpart of current disability measurements and some of our results (eg, standardized movement performance) apply only to sitting movements. While the specific results offered in this paper give insight into usage and perception that are unlikely to change dramatically, the quality of the data produced needs to be continually assessed. New techniques to continually evaluate a system as changes are introduced are needed to provide sustained evidence of usability [22].

In addition, no testing was performed with participants with severe visual or hearing loss, although we would expect the health professional to play a mediating role in these situations if extra help is required.

### Comparison With Previous Work

There are a growing number of computer-assisted and sensor-based applications that support clinical assessment and rehabilitation for MS patients. These include social gaming [23], exoskeletons [24], and virtual environments [25]. More recently, a number of depth-sensing computer vision applications for MS rehabilitation have also been evaluated (eg, [7]), which show both successful implementation and good acceptability by patients.

Sensor-based recordings of human movements are becoming increasingly important for assessment of symptoms in different neurological disorders in addition to the strides made in rehabilitation [26]. In MS specifically, body-worn motion sensors can detect mobility difference between healthy volunteers and patients with early stage MS better than traditional time tests [27]. Accelerometers have also been used to measure both physical activity and walking mobility in MS patients [28]. Most recently, an accelerometer built into an iPad has been used for gait and balance analysis [29]. There are also initial findings about the use of depth sensing for carrying out gait analysis in MS patients [30]. Sensing technology to support patients with other conditions that cause motor dysfunction is also being developed [31].

Despite initial research in the area, applications that use sensing (vision or other) for clinical assessment have not been deployed in clinical settings. This study shows that attention to the clinical environment in the design process can make these new approaches to medicine a reality.

### Conclusions

Depth-sensing computer vision has been rapidly adopted to form the core of a range of innovative health care applications for the clinical assessment and rehabilitation of movement ability. There are now increasing numbers of examples of the commercialization of such ideas for rehabilitation. Yet, clinical assessment has been a greater challenge, with the need for greater precision of measure, and hence lower variability in the data.The creation of ASSESS MS, as part of one of the first projects in this domain, shows that careful attention to deployment makes it possible to collect sensor data of a quality needed for clinical assessment. Moreover, it can be done in a way that is suitable to wide-scale deployment and acceptable to patients. These results open the door for greater development in depth-sensor-based assessment of movement disorders.

## Appendix

Multimedia Appendix 1 ASSESS MS training materials.

Multimedia Appendix 2 Questionnaire and interview materials.

Multimedia Appendix 3 Standardized movement performance rating guide.

## Abbreviations

EDSS: Expanded Disability Status Scale
MS: multiple sclerosis
SDMT: Symbol Digit Modalities Test
