# Supplementary material for: Usability and Acceptability of ASSESS MS: Assessment of Motor Dysfunction in Multiple Sclerosis Using Depth-Sensing Computer Vision
Source: JMIR Hum Factors. 2015 Jun 24;2(1):e11. doi: 10.2196/humanfactors.4129 (PMC4797664; doi:10.2196/humanfactors.4129)
Supplement: Multimedia Appendix 1 [file humanfactors_v2i1e11_app1.pdf]

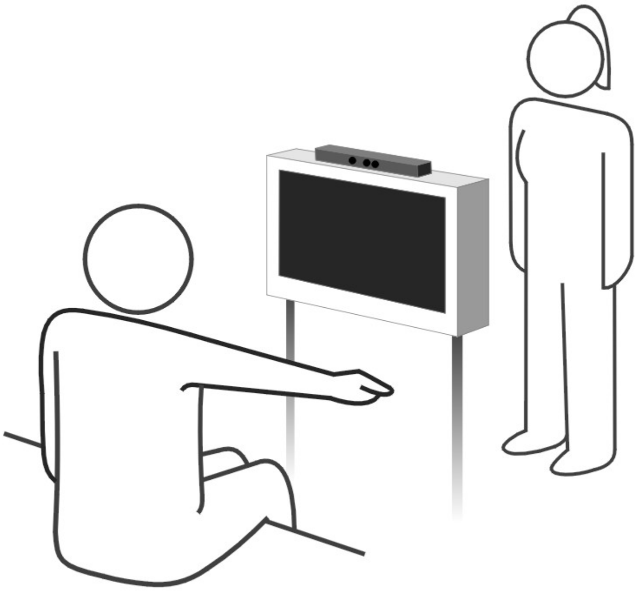

# ASSESS-MS training protocol

CPM/KH 2014

This revised protocol reflects learning from 7 training sessions given as part of the ASSESS-MS UI study conducted in August 2014.

## Session preparation

- Set up the ASSESS-MS device, check that the remote control is working and navigate to the initial positioning screen (use patient number 88888) so that the Kinect output is visible from the start of the training. Find a polished metal object that can simulate distracting reflection.
- The session includes a simulated ASSESS-MS examination, so the setting must be configured appropriately with an examination bench, table with cup of water and book, and space for the standing/walking examinations.
- Prepare copies of the movement protocol summary (Appendix to this protocol) to give to trainees.
- Load the training presentation onto a laptop or other display (better not to use the ASSESS-MS tablet) ready to be shown at the end of training. Review the presentation and the associated notes.

## Outline of session

- Training takes roughly 1 hour and is split into four parts:
    1. Conceptual introduction (3 minutes)
    2. Simulated examination (45 minutes)
    3. Patient management aspects (2 minutes)
    4. Pitfalls (10 minutes)
- The session can be extended by giving trainees an opportunity to practice at the end.
- The recommended number of trainees is 2-3.

## 1. Conceptual introduction

- Introduce ASSESS-MS: a ‘talking camera’ system intended to support the consistent measurement of movement ability for clinical trials by capturing a series of movements performed by a patient.
- Explain the two issues, relevant for clinical trials, that the device is trying to solve:
    - High inter- and intra-rater variability when assessments are performed by humans. High score variance increases the sample sizes required to demonstrate improvements in trials and may also threaten the validity of inferences based on human-assigned ratings.
    - Limited granularity of the resulting scores that makes it hard to detect subtle changes in disease state. Detecting small improvements, sooner, would be useful for clinical trials that currently have to run over many months.
- Explain how the system aims to address these issues by offering a reproducible, standardised way to capture movement and analysis techniques that can identify subtle changes in disease.
- Explain the components of the system:
    - Kinect camera, which can track movement in three dimensions. Illustrate effect on positioning screen by passing hand in front of camera. Explain colouring scheme for depth (from close to distant: black>red>green>grey>red).
    - Talking screen for patients
    - Touch screen panel for clinicians.

## 2. Simulated examination

- Explain that the easiest way to get to know the system is to walk through an examination. Ask for a volunteer and get them to sit on the examination couch.
- Give movement protocol handouts to trainees.
- Orient ASSESS-MS so that both patient and clinician screens are visible to trainees, including trainee taking part in examination.
- Provide a conceptual overview of the examination, with a flow that goes from seated to standing:
  - 6 sitting movements, including two functional movements
  - 3 standing movements
  - 2 walking movements

### 2.1 Positioning screen

- Explain principles of positioning seated patient (the trainee):
  - Patient should be centred, using graticule on positioning screen
  - Outstretched arms should be contained within dotted bounding box
- Highlight availability of quantitative distance measure (**highlight that intended distances are listed on handouts – 1.9m for seated**) but that this should be secondary to common sense about framing the patient.

### 2.2 Introduction to navigation

- Once positioning is satisfactory, explain principles of navigating using touch interface:
  - Option to press the Done button on the positioning screen, or
  - Using the navigation arrows at the top of the screen.
- Explain that arrows are always available to move forwards and backwards as required. Explain that this can be used to skip examinations if they cannot be performed at all.
- Explain that the interface is very sensitive and a light touch is all that is needed.

### 2.3 Introduction/distractions screen

- Explain that there is a video that you should show the patient that summarises the principles of the system. **Play the video.**

- State that the clinician screen shows a different view and this is of things that are distracting the camera. Explain briefly how shiny things interfere with how Kinect sees depth. Illustrate the effect on the positioning screen using a shiny object and the principle of eliminating objects framed in green.
- Discuss common potential sources of distraction (e.g. wheelchairs, crutches) and strategies for removing them with the patient's permission.

## 2.4 Introduction to examination interface

- Advance to the TAT introduction screen.
- Explain, and illustrate, how each test is divided into two parts:
  - An introductory screen with a video for the patient. The screen also has a small positioning display which should be checked periodically to make sure the patient is still centred.
  - One or more test screens to actually perform the test. These are accessed by tapping Begin. Some tests have multiple variants – for example eyes open or eyes closed.
- Explain that the trainee doesn't need to worry about remembering all the variants, the system will automatically step through them.

## 2.5 Performing an examination (TAT)

- Explain, **and perform**, the standard sequence common to all the tests, using TAT as the example:
  - Preparation: explain that for TAT feet need to be off the floor. Show how this information is displayed on the clinician screen (and on the handout).
  - Play the introductory video. Explain that this can be repeated as many times as required.
  - Briefly check patient's understanding.
  - Ensure that the patient is in the neutral starting position. **This is always, for seated movements, with hands on lap.**
  - Click Begin and illustrate how the test variant is clearly shown (eyes opened for TAT).
  - Press Record to start the examination. Emphasise the importance of not starting the movement until the beep sounds and the screen border goes red.
  - Explain how the test must be performed for 5 seconds. **Timing is up to the clinician**, although the system provides a timer.

  - Press Record again to stop the examination.

- Emphasise the simplicity of the process: the simple sequence that was just completed is the same for all the examinations.

- Show how the user interface has automatically advanced to the next variant of the TAT test (eyes closed). **Introduce the progress bar.**

- Explain, **and show**, what to do if the recording needs to be repeated. Explain that patients are often keen for their first test and it is easy to miss the start of the first movement. Discuss the balance of repeating tests to get them right but also being sensitive to patients if they are having difficulty understanding the instructions.

## 2.6 Seated examinations

- Step through the seated examinations, playing the introductory videos and highlighting salient points about their conduct (also on handout). Perform the examinations as required (not necessary for all variants).
  - TAT
    - **Feet must be off floor – raise bed.** Most people forget this when they start performing examinations. Raising the bed exposes truncal instability.
    - Up to clinician to count to 5 seconds and then tell the patient to put their arms down
    - Both eyes opened and eyes closed in separate tests
  - FFT/FNT
    - **Feet should be on floor – lower bed.** Truncal instability would compromise this and subsequent seated tests.
    - Arms stretched outwards, bending at the elbow horizontally
    - 3 repetitions, each ending with arms extended outwards. Emphasise that the computer cannot count the repetitions.
    - Both eyes opened and eyes closed in separate tests
  - DRS
    - Direction movement is always **down and out** to start
    - Only four sides of a square, not five.

    - Both together then each side, both eyes opened and closed
- TPF/TPB
    - Need table and notebook
    - Placement of book centred in front of patient, opened to the middle
    - Turn three pages either forward or backwards without returning hands to lap between each page.
- CUP
    - Cup should be three quarters full, refill for other side if less than half full
    - Position at arm’s length in front of relevant shoulder
    - **Two sips** each side, moving the cup to the mouth, not the head
    - Place arms back in lap **after each sip**

## 2.6 Standing and walking examinations

- Address positioning for standing/walking (2.8 and 5m distances, respectively).
- Address importance of protecting patient against falling and possibility of omitting tests if patient is unable to stand/walk at all.
- Introduce remote control as a way of ensuring clinician can stay with patient to support them if necessary. Explain clinician should always stand and travel on patient's left.

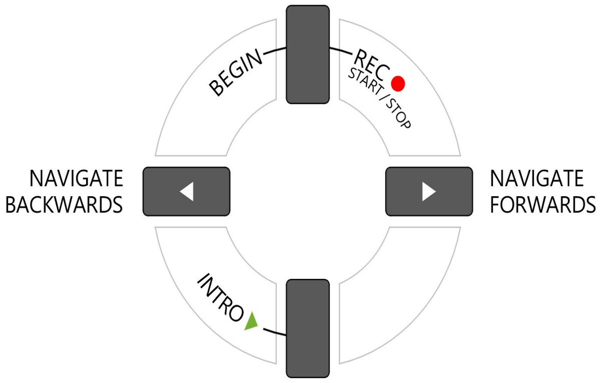


- Address importance of protecting patient against falling and possibility of omitting tests if patient is unable to stand/walk at all.
- Step through examinations:
  - ROM
    - Introduce as the single most complicated examination because the introduction provides little guidance
    - Feet together, arms up to shoulder height, palm up but only when started
    - Up to clinician to count to 5 seconds for each of eyes opened and closed
    - Examiner should be on the patient's left side to support in case of imbalance
    - Examiner briefly raises arm as patient switches to eyes closed
  - RSA
    - One full turn towards the side indicated
  - HOP
    - Should be 45 degrees with hopping side nearest camera and clinician in front

    - Supported by holding hands. Emphasise that patient safety is a priority, but the camera needs to have a clear view of the patient.
    - 10 hops, or as many as possible, with **eyes open**
  - GAT
    - Must be unaided
    - Stand by to support patient if necessary on the patient’s left hand side
    - Walk towards the camera, then back and then towards the camera again
  - TRW
    - Unlike GAT, only need to walk to the camera once.

## 3. Patient management

- Navigate to the Start screen and explain how to initiate an examination by pressing Start.
- Walk through the patient data screen.
    - Explain how patient, location and room numbers will be provided.
    - Explain data validation and consequences of incorrect data entry (box is highlighted red, cannot advance to next screen)
- Advance to positioning screen which is where training started

## 4. Pitfalls

- Use the pitfalls PowerPoint presentation to recap and highlight common issues. The presentation notes detail topics that could be addressed.

# Movement Protocol

SIX SITTING MOVEMENTS   (1.9 metres)

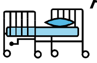All movements begin and end with the hands in the lap.

1. Ataxia

Stretch out the arms to the side for **five seconds**.

- The arms should be as straight as the patient is able to make them.
- 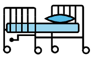This test is done with the feet OFF the ground: **raise the bed**

2. Finger to Nose Test

Stretch out the arm to the side and touch the nose **three times** ending.

- The arm should start to the side (NOT the front).
- This test is done with feet ON the ground: **lower the bed**

3. Finger to Finger Test

Stretch out the arm to the side and touch the nose **three times** ending.

- The arm should start to the side (NOT the front).

4. Drawing Squares

Draw two imaginary squares. Start with the hands at eye level, draw down, out, up, and in.

- Start DOWN, and OUT
- Draw four sides of a square only

5. Turning Pages Forward and Backwards

Turn 3 consecutive pages forward in the text of the book with the left hand.

- Place a notebook in front of the patient.

6. Drinking From a Cup

Reach out and grasp the cup, take a sip, puts the cup down, puts the hand back in the lap. **Repeat.**

- Place a small table in front of the patient and place a cup of water that is ¾ full in at arm’s length in front of shoulder.

THREE STANDING MOVEMENTS (2.8 metres)

7. Romberg

Put the feet together, lift arms to shoulder height and turn palms up. Hold for 5 seconds and then ask patient to close their eyes for another 5 seconds. **Stand to the patient's left.**

- Wave at camera when patient closes eyes

8. Rotating on the Spot

Turn on the spot in the direction shown.

9. Hopping on One Foot

The patient turns on a forty-five degree angle and hops 10 times on the foot closest to the camera.

- Stand to the patient's left the first time and to their right the second time.

TWO WALKING MOVEMENTS (5 metres)

10. Normal Walking

Walk to the camera, turn around and walk back to the starting position, and turn again and walk towards the camera. **Stand to the patient's left.**

11. Tightrope Walking

Walk towards the camera, placing one foot in front of the other.

# ASSESS-MS Training Reflections

Basel August 04/08, Bern August 11/12, Amsterdam August 19/20 (KH + 13 clinicians). Over the course of six training sessions introducing clinical staff to the ASSESS-MS system, KH had a chance to refine the way in which the principles of ML underpinning the system operation were explained. The motivation for this explanation was to reinforce messages about the importance of movement conduct standardisation that was a main focus of the training process.

The same two videos were used to assist explanation, one showing a single depth video, described as "An example of the one of the videos that the trainees had just captured", and a composite video showing the same movement performed by multiple individuals, described as "How the system sees the world."

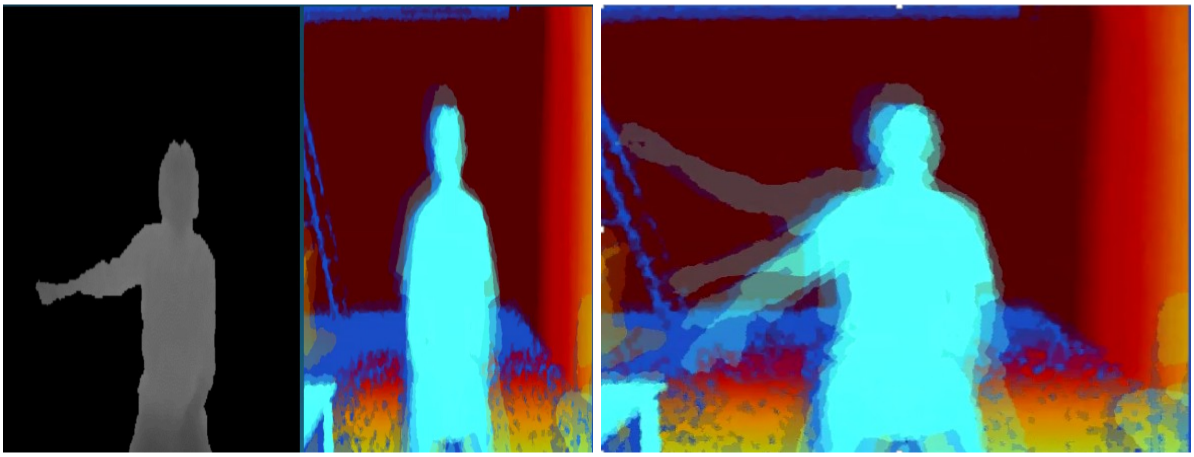

Over the training sessions KH attempted to actively refine the explanation in response to whether, and how easily, each of the trainees appeared to grasp the concepts being communicated. Strategies that appeared to be less successful were:

- Using the second panel to explain that the computer had learned the movements of people with a wide range of functional ability; that consequently it knew what it is that people with a score of zero look like, what it is that people with a score of one look like, and so on, and therefore when given data from someone new it can use this information to place them in the appropriate group. This explanation requires an abstraction – the idea that there are characteristics of those with a particular score – which did not appear to resonate with trainees. Nor did it directly address our emphasis on the need for standardisation.
- Using the first panel to build agreement with the trainees that it is easy for a human to interpret movement because of our familiarity with what normal motion looks like at both an intuitive and a more explicitly descriptive level. We can attend to specific

features that fit with our conceptual understanding of how dysfunction can affect the movement, whereas for a computer there would be no sense of ‘normal movement’ other than that derived from what it has seen of healthy and unhealthy movements, illustrated by the variation seen in the composite second panel.

The most successful strategy involved a highly simplified conception of the ML process:

- That the ML functions simply by asking the question “Which of these many patients that I have learned from in the past does this new patient most look like?” This was particularly successful in respect of reinforcing the need for standardisation because of the consequence of this form of explanation: “If a patient looks like score X then they will be labelled as X. This doesn’t matter if they look like X because they actually have dysfunction or they look like X because they didn’t perform the movement correctly.” The four trainees offered this explanation appeared to easily grasp their role, and that of the supporting UI, in trying to avoid the second contingency, often completing the sentence as it was being said and nodding in agreement: “We need to make sure when they can’t do a movement right, it’s because the patient really can’t do it, not because they didn’t ‘get it’.”
