# Supplementary material for: Usability and Acceptability of ASSESS MS: Assessment of Motor Dysfunction in Multiple Sclerosis Using Depth-Sensing Computer Vision
Source: JMIR Hum Factors. 2015 Jun 24;2(1):e11. doi: 10.2196/humanfactors.4129 (PMC4797664; doi:10.2196/humanfactors.4129)
Supplement: Multimedia Appendix 2 [file humanfactors_v2i1e11_app2.pdf]

Study Questionnaires

Health Professional Questionnaire

Likert Scale Questions

1. The recording system was easy to use.
2. I would use the recording system in future examinations.
3. The movement instructions given by the recording system were clear to the patient.
4. I prefer to demonstrate the movements to the patient myself.
5. Using the recording system during the examination made me feel awkward or uncomfortable.

Patient Questionnaire

Likert Scale Questions

1. I understood what to do during the study examination.
2. I would not like my health professional to use the recording system during my future examinations.
3. The movement instructions given by the recording system were clear.
4. I prefer my health professional to demonstrate the movements.
5. Using the recording system during the examination made me feel awkward or uncomfortable.

Free Response Questions

1. Please write down three words that you would use to describe the recording system to another patient.

2. What was most helpful about the recording system in performing the study examination?

3. What would you most like to change about the recording system?

4. Further comments (optional).

Health Professional Interview Guide

1. Please give three words that you would use to describe the recording system to another health professional.

2. How does the VMCS study examination differ most from other examinations that you do in your daily routine?

3. What was most helpful about the recording system in performing the study examination?

4. What would you most like to change about the recording system?

5. Were there any parts of the User Interface that you found confusing?

6. Were there any parts of the User Interface that you would not want changed?

7. Further comments (optional).
