# Supplementary material for: Usability and Acceptability of ASSESS MS: Assessment of Motor Dysfunction in Multiple Sclerosis Using Depth-Sensing Computer Vision
Source: JMIR Hum Factors. 2015 Jun 24;2(1):e11. doi: 10.2196/humanfactors.4129 (PMC4797664; doi:10.2196/humanfactors.4129)
Supplement: Multimedia Appendix 3 [file humanfactors_v2i1e11_app3.pdf]

Movement Performance Scoring

visitName
movie name
Compliance of Movements
**Finger to Nose**
a. Hand stretched to side
b. Correct number of repetitions (3 times)
**Finger to Finger**
a. Hands travel horizontally
b. Correct number of repetitions (3 times)
**Ataxia**
a. Arms are straight as able
**Drawing Squares**
a. Correct direction
b. Correct number of square sides
**Drinking from a Cup**
a. Hand returns to lap before repetition
b. Correct number of repetitions (twice)
